# The Impact of Obesity on Hodgkin’s Lymphoma Patients Treated With Uniform Chemotherapy Protocol at Princess Noorah Oncology Center, National Guard Health Affairs, Jeddah, Saudi Arabia: Retrospective Matched Cohort

**DOI:** 10.7759/cureus.25002

**Published:** 2022-05-14

**Authors:** Alaa T Alsharif, Mohammed Aldawsari, Emad M Babateen, Meshaal A Kouther, Faisal F Aljahdali, Ahmed Absi, Taghreed Aldosary, Mohamed E Ahmed

**Affiliations:** 1 College of Medicine, King Saud Bin Abdulaziz University for Health Sciences, Jeddah, SAU; 2 Hematology/Oncology/Bone Marrow Transplant, Princess Noorah Oncology Center, King Abdulaziz Medical City, Ministry of National Guard Health Affairs, Jeddah, SAU; 3 Medical Sciences/Oral Biology, King Abdulaziz Medical City, Ministry of the National Guard Health Affairs, Jeddah, SAU; 4 Biostatistics, King Abdullah International Medical Research Center, Jeddah, SAU

**Keywords:** obesity and hodgkins lymphoma, diabetes, chemotherapy, abvd, obesity, hodgkin lymphoma

## Abstract

Background

Hodgkin's lymphoma (HL) is a disease that affects lymphocytes, mostly B cells, and it is commonly diagnosed by the presence of Reed-Sternberg cells. The influence of obesity on the disease course of HL is still controversial. This study’s aim was to investigate the treatment outcomes in obese patients suffering from HL and compare them to the outcomes of non-obese patients.

Methods

This study is a single-center retrospective cohort study that included 280 patients admitted between 2009 and 2020 with different subtypes of HL who received the chemotherapy regimen of Adriamycin, bleomycin, vinblastine, and dacarbazine (ABVD) at Princess Norah Oncology Center, National Guard Hospital, Jeddah, Saudi Arabia. Based on WHO criteria, the participants were divided into two groups (obese with a BMI that exceeds 30 kg/m2 versus non-obese with any BMI less than 29,9 kg/m2). All demographic data including age, gender, BMI, body surface area (BSA), and HL subtype (nodular sclerosis, mixed cellularity, lymphocyte depletion) were recorded. In addition, the presence of diabetes mellitus (DM), previous cancer, smoking, staging of HL, number of cycles of ABVD, dose intensity of ABVD, and outcomes (emergency visits, death during therapy, primary resistance, relapse) were collected from the participant files.

Results

Regarding therapy outcomes, 24.1% of obese patients were admitted to the hospital after receiving the first cycle of ABVD as compared to 75.9% of non-obese patients. However, there was no significant statistical difference between obese and non-obese patients in their hospital admission (p value=0.500). In addition, non-obese patients had a higher chance of being admitted to the hospital after receiving the chemotherapy dose with an odds ratio of 1.22 compared to obese patients. For the emergency visits, 20.8% of obese patients were admitted to ER as a complication of the chemotherapy regimen, whereas 79.3% of non-obese patients were admitted to ER after receiving the chemotherapy. The P-value was statistically not significant (0.396), but the odds of ER admissions after ABVD cycles were 1.28 times higher in non-obese patients compared to obese.

Conclusion

The study outcomes showed a higher odds of hospital admission and ER admission as complications of the chemotherapy regimen in non-obese HL patients as compared to obese patients.

## Introduction

Hodgkin's lymphoma (HL) is a disease that affects lymphocytes, mostly B cells, and it is commonly diagnosed by the presence of Reed-Sternberg cells [1]. The chemotherapy regimen that is commonly administered to patients with HL includes Adriamycin, bleomycin, vinblastine, and dacarbazine (ABVD) [1]. In 2016, the Saudi Health Council reported that the incidence of HL in Saudi Arabia is 3.8% [2]. However, according to the American Institute for Cancer Research, the incidence of the disease worldwide is 0.5% [3]. Since the five-year survival rate of the disease exceeds 90%, accordingly, most of researchers focus on complications and long-term effects from chemotherapy [1,4]. Meyer et al. reported that out of the 15 deaths that they encountered during their study, six were attributed to HL and acute toxicities, and nine were due to complications of chemotherapy [4]. Schwenkglenks et al. stated that one of the main side effects and complications of anticancer chemotherapy is myelosuppression that might cause a life-threatening febrile neutropenic episode [5]. The prevalence of obesity in Saudi Arabia is high and the rate hit 28.7% in adults [6]. Moreover, WHO estimated In 2016 that obesity is seen in about 13% of the world’s adult population [7]. Based on the criteria of WHO, it is estimated that normal-weight individuals have a BMI between 18.5 and 24.9 kg/m2; overweight individuals have a BMI between 25 and 29.9 kg/m2, and obese individuals have a BMI that exceeds 30 kg/m2 [6].

It has been reported in many studies that there is an association between BMI and cancer outcome. Pan et al. reported that obesity and BMI have been identified as risk factors in development of cancer [7]. Another study demonstrated that BMI was an independent prognostic factor for overall survival in multivariate analysis with P-value < 0.014 [8]. Obesity's influence on the disease course of HL is still controversial. Nonetheless, Landgren et al. reported that BMI was independently and significantly associated with cause-specific survival (CSS) [9]. In addition, they claimed that the corresponding five-year overall survival (OS) for the BMI groups was 100% (obese), 79% (overweight), and 70% (normal and underweight), respectively [9]. They found that in HL patients treated with chemotherapy, high BMI was significantly associated with a better prognostic risk profile and improvement in CSS [9]. Preston et al., 2017; stated that there was no significant difference in the level of neutropenia between high BMI and low BMI groups diagnosed with HL at the end of their treatment [10]. Nevertheless, Sheng et al. stated that adipocytes sequester and metabolize the chemotherapeutic drugs [11]. Pergola et al. reported that patients in the group of the highest BMI quartile largely expressed markers of high proliferation of the tumor cells as compared to those with similar-size tumors in the non-obese [12]. Willett et al. and Boyle et al. reported that a high BMI increased the risk of HL more than two-fold compared to those in the normal BMI [13,14]. Lichtman et al. stated that “the metabolic, endocrinologic, immunologic, and inflammatory-like changes resulting from obesity may increase the cell mutation rate, dysregulate gene function, disturb DNA repair, or induce epigenetic changes, favoring the induction of neoplastic transformation” [15]. Finally, Ligibel et al. claimed that obesity has been associated with an increase in the risk of toxicities from chemotherapy, including fatigue, and neuropathy [16].

HL is a highly curable malignancy that affects relatively young populations and is not uncommon in Saudi Arabia. On the other hand, obesity is a major public health challenge in Saudi Arabia. While researchers have explored the association between both problems in terms of treatment outcomes and chemotherapy toxicities, no study in Saudi Arabia has been done on HL patients to examine the association between BMI level and the chemotherapy outcomes, and this highlights the importance of our research. Our aim is to assess the impact of obesity on HL patients treated with uniform chemotherapy protocol (ABVD) at Princess Norah Oncology Center, National Guard Health Affairs, Jeddah, Saudi Arabia.

## Materials and methods

It is a single-center retrospective cohort study that included 230 patients (obese versus non-obese) admitted with different subtypes of HL who received the chemotherapy regimen ABVD at Princess Noorah Oncology Center, National Guard Health Affairs, Jeddah, Saudi Arabia, between 2009 and 2020. We retrospectively went through the patient’s files from multiple systems and the older patient files and looked for our variables from them and inserted the data in an Excel sheet (Microsoft Corporation, Redmond, Washington, United States). After fully inserting the data, we analyzed the findings to get the results. Data were collected using BESTCare (ezCaretech Co, South Korea), Chemo Rx system (Saudi Ministry of the National Guard, National Guard Health Affairs, Saudi Arabia), ARIA (Aria Systems, San Francisco, California, United States), and older records of patients and were inserted in a data collection sheet in an encrypted Excel file, filled only by the members of the research using a password-protected personal computer.

We included all patients who were admitted to our oncology center with an established diagnosis of HL (based on clinical features, including B symptoms, lab finding including complete blood count (CBC), imaging finding including CT scan and positron emission tomography (PET) scan, and lymph node biopsy) and received the chemotherapy regimen ABVD. Patients who were diagnosed with nodular lymphocyte-predominant HL and those who had active cancer at the time of diagnosis of HL were excluded. All demographic data including age, gender, BMI, body surface area (BSA), HL subtype (nodular sclerosis, mixed cellularity, lymphocyte depletion), presence of diabetes mellitus, previous cancer, smoking, staging of HL, number of cycles of ABVD, dose intensity of ABVD, outcomes (emergency visits, death during therapy, primary resistance, relapse) were recorded.

Statistical analysis was performed by the use of IBM SPSS Statistics for Windows, Version 20.0 (Released 2011; IBM Corp., Armonk, New York, United States). Descriptive statistics were carried out by reporting the number and percentage for categorical variables, and the mean and standard deviation for continuous ones. Demographic and clinical data as well as close-ended questions were summarized in frequency tables. In the comparison between obese HL and non-obese HL, the chi-squared test was used for categorical variables, while the t-test was used for continuous ones. For this, adjusted odds ratio (OR), and 95% confidence interval (CI) were reported, with the level of significance taken as P-value <0.05. For survival analysis, the Kaplan-Meir estimate and Cox Regression model were conducted.

## Results

Data were taken from 280 patients with mean age of 36.2 years showing that 143 (51.1%) were males and 137 (49.9%) were females. In the aspect of diabetes mellitus at the beginning of therapy, 26 patients (8.9%) had been diagnosed with diabetes mellitus while 254 patients (91.1%) had not. In addition, 42 patients (15%) were smokers. Moreover, the conducted data revealed that just four patients were diagnosed with previous cancer. Finally, 80 patients (28.9%) were on the limited protocol and 200 patients (71.1%) were on the advance protocol as shown in Table 1

**Table 1:** Basic characteristics of patients DM: diabetes mellitus

Variable	N=280	%
Gender		
Male	143	51.1
Female	137	49.9
DM at beginning of therapy		
Diagnosed with DM pre-treament	26	8.9
Not diagnosed with DM pre-treament	254	91.1
Smoker		
Yes	42	15.0
No	238	85.0
Previous cancer		
Yes	4	1.4
No	276	98.6
Limited or advanced		
Limited	80	28.9
Advanced	200	71.1
Age	Mean 36.2	SD 15.8

The BMI at baseline was 25.2 (kg/m2) with SD 7.02. This reflects that the average of our patients was overweight (based on the BMI classification) at the beginning of therapy. The mean of the BSA baseline was 1.78 with SD of 0.85. the dose of the therapy was depending on BSA. The mean BMI at the end of therapy increased but remained in the same category as the baseline with an SD that was significantly increased in comparison to the SD at baseline. The mean of the last recorded BMI was 28.1, lower than that of the end-of-therapy BMI, as shown in Table 2.

**Table 2:** Diagnosis characteristics BSA: body surface area

Variable	Mean	SD
Height (cm)	162.5	14.0
Baseline therapy Weight (kg)	67.5	20.5
BMI at baseline (kg/m2)	25.2	7.02
BSA at baseline	1.78	0.85
End-of-therapy weight (kg)	71.2	19.5
Last weight (kg)	73.4	19.9
End-of-therapy BMI (kg/m2)	28.7	21.2
Last recorded BMI (kg/m2)	28.1	8.4

With regard to therapy outcomes, 17.9% of obese patients were admitted to the hospital after receiving the first cycle of ABVD compared to 22% of non-obese patients. There was no significant statistical difference between obese and non-obese patients in their hospital admission (p value=0.500). However, the odds ratio reveals that non-obese patients are more likely to be admitted to the hospital after receiving the chemotherapy dose compared to obese patients. For the emergency visits, 28% of non-obese patients were admitted to ER as a complication of chemotherapy regimen, whereas 22% of obese patients were admitted to the ER after receiving the chemotherapy. Though the p-value was statistically not significant (0.396), the ER admissions after ABVD cycles was higher in non-obese patients compared to the obese. Furthermore, only two deaths were recoded among 280 patients of HL after receiving the chemotherapy dose. There was no significant statistical difference between obese and non-obese patients in their remission outcome (p-value=0.219); however, odds ratio suggests that the obese patients are more likely to be in remission compared to non-obese patients (OR=1.56). For the relapse as outcome, patients who relapsed comprised 15.4% obese patients and 19% of non-obese patients. Though there was no significant statistical difference between obese and non-obese patients in their relapse outcome (p-value=0.729), obese patients have higher chances to relapse compared to non-obese patients (OR=0.846). Among those developing diabetes mellitus type 2 at the end of the therapy as an outcome, 6.4% of the cases were obese compared to 7% of non-obese patients, and there was no significant statistical difference (p-value=0.922). Odds ratio indicates that obese patients are less likely to develop diabetes mellitus type 2 at the end of the therapy (OR=0.936) as shown in Table 3.

**Table 3:** Association between obesity and the outcomes of therapy DM: diabetes mellitus; CR: complete remission; PR: partial remission Remission is defined as "a decrease in or disappearance of signs and symptoms of cancer. In partial remission, some, but not all, signs and symptoms of cancer have disappeared. In complete remission, all signs and symptoms of cancer have disappeared, although cancer still may be in the body" [17].

Outcome	n	%	P value	OR
Hospital admission after first cycle	94	33.6	0.500	0.821
Emergency visits	97	34.6	0.396	0.78
Death during therapy	2	0.7	0.444	-
Primary resistant	14	5.0	-	-
Relapse	30	10.7	0.729	0.846
DM at end of therapy	8	3.4	0.922	0.936
Remission			0.219	1.56
CR	215	76.8		
PR	65	23.2		

## Discussion

Princess Noorah Oncology Center is the largest oncology center in the western region of Saudi Arabia; hence, it receives most HL cases indicated for chemotherapy and advanced clinical management. In addition, the association between obesity and HL patient response to ABVD regimen is still controversial. These two factors raised the idea of the current study. No similar study has been done in Saudi Arabia comparing obese and non-obese HL patients in terms of their response to chemotherapy regimens and this signifies the importance of our research.

Pan et.al. reported that obesity increases the risk of overall cancer, non-HL, leukemia, multiple myeloma, and cancers of the kidney, colon, rectum, breast (in postmenopausal women), pancreas, ovary, and prostate [7]. Their observations give strong evidence for the positive association between obesity and overall cancers [7]. They found a positive association with not only obesity but also the overweight category in both sexes. For the association of obesity and mechanisms of several type of cancers they found that the mechanism of non-HL, leukemia, and multiple myeloma is unclear. However, it could be due to the decreased immune response associated with obesity and a lower intake of antioxidants and other nutrients [7].

In addition, Dobbins et al. stated that a significant, positive, and, for some cancers, a strong association between obesity and cancer incidence. Given that a significant proportion of cancer could be avoided if obesity was eliminated or significantly reduced, they found that certain types of cancer were far more likely to develop in obese people like renal cancer for men, with a relative risk of 1.57 and gallbladder cancer for women with a relative risk of 1.82 [18].

In terms of the association of obesity with HL, Strongman et al. studied the nature of this relationship in individuals aged 16 and older by using the data of primary care from the United Kingdom’s Clinical Practice Research Datalink. The results showed that each 5k/m2 increase in BMI was correlated to an increase of 10% in the development of HL (95%CI 2-19). The analysis of non-linearity shows a j-shape relation to increasing the incidence with a BMI higher than 24.2k/m2 [19]. Furthermore, Kanda et al. conducted a case-control study in a Japanese population that included 782 cases of malignant lymphoma and concluded that the increase in recent weight and BMI showed a slightly significant association with malignant lymphoma risk (ORs (95% CIs) per five‐unit increase in recent weight and BMI; OR 1.04 (95%CI 0.99-1.09) and OR 1.11 (95%CI 0.98-1.27), respectively [20].

Our study found that obese HL patients are less likely to be admitted to the hospital and ER compared to normal-weight patients. Moreover, obese HL patients are less likely to develop type 2 diabetes and are more likely to be in remission compared to non-obese HL patients. These findings are in line with Landgren et al. who reported a five-year OS, which was 100% for patients treated with chemotherapy, classified as obese pre-treatment [8].

Hourdequin et al. performed a systematic review of studies that compared survival outcomes and toxic effects of chemotherapy between obese and non-obese patients based on their actual body weight. This review included 9314 patients and concluded that there was no statistical difference between obese and non-obese patients in terms of the outcomes after receiving the chemotherapy dose [21].

Limitations

There was no statistical significance in the outcome parameters, and this is attributed to the huge difference between the number of non-obese HL patients compared to the number of obese HL patients. We collected the data from 280 patients, where 50 of them were obese, which accounts for about 20% of cases, and the remaining were non-obese, which accounts for 80% of cases. We considered a patient with HL to be obese based on his/her BMI being above 30 kg/m2 without considering the overweight category; hence, that limited our patients’ sample size. Furthermore, the outcome parameters that we relied on in our study were confined to ER visits, hospital admission, remission, and relapse, which were not specific to determining the outcome of ABVD administration.

## Conclusions

Our study revealed no statistical significance in the outcome parameters, and this is attributed to the huge difference between the number of non-obese HL patients compared to the number of obese HL patients in our study. However, the odds ratio indicated that obese HL patients are less likely to be admitted to the hospital and ER compared to non-obese patients. Moreover, obese HL patients are less likely to develop type II diabetes. However, obese HL patients are more likely to be in remission compared to non-obese HL patients.
